# Latent multimorbidity classes and antithrombotic medication distributions among patients hospitalized for bleeding after dental surgery: a retrospective study

**DOI:** 10.3389/fsysb.2026.1917159

**Published:** 2026-09-24

**Authors:** Juliane Wagner, Johannes Heinrich Spille, Jill Flor, Zeyue Ouyang, Jonas Conrad, Christof Dörfer, Jörg Wiltfang, Hendrik Naujokat

**Affiliations:** 1 Department of Oral and Maxillofacial Surgery, University Hospital Schleswig-Holstein, Campus Kiel, Kiel, Germany; 2 Cluster of Excellence, Precision medicine in inflammation, Christian-Albrechts-University of Kiel, Kiel, Germany; 3 Clinic of Conservative Dentistry and Periodontology, University Hospital Schleswig-Holstein, Campus Kiel, Kiel, Germany

**Keywords:** anticoagulation, direct oral anticoagulants, latent class analysis, multimorbidity, oral surgery, postoperative bleeding, vitamin K antagonists

## Abstract

**Objectives:**

To characterize patients hospitalized for postoperative intraoral bleeding after dental surgery and to describe latent multimorbidity classes and their observed antithrombotic medication distributions.

**Materials and Methods:**

This single-center retrospective study included 700 patients hospitalized between 2011 and 2021. Demographic characteristics, clinical measurements, comorbidities, and antithrombotic medication categories were extracted from medical records. Latent class analysis was performed in 458 patients with at least two documented comorbidities using 11 binary indicators. Models containing one to six classes were compared using information criteria, entropy, the bootstrap likelihood ratio test, class size, and clinical interpretability. Stability of the selected solution was assessed using 100 bootstrap resamples.

**Results:**

The median age was 74 years, 460 patients (65.7%) were male, and 581 (83.0%) were receiving at least one antithrombotic medication. A five-class solution was selected. Class 1 (n = 26, 5.7%) was characterized by deep venous thrombosis, hypertension, and cardiac disease; Class 2 (n = 97, 21.2%) by heart valve replacement and cardiac disease; Class 3 (n = 55, 12.0%) by stroke, cardiac disease, and hypertension; Class 4 (n = 151, 33.0%) by hypertension and cardiac disease; and Class 5 (n = 129, 28.2%) by a broader multisystem profile involving cardiac disease, hypertension, diabetes, renal disease, and gastrointestinal disease. Vitamin K antagonist (VKA) use was most frequent in Classes 1 and 2, whereas direct oral anticoagulant (DOAC) use was most frequent in Class 4. Bootstrap analysis indicated moderate overall stability.

**Conclusion:**

The study describes clinically relevant heterogeneity among hospitalized bleeding cases.

## 1 Introduction

Older patients undergoing dental and oral surgical procedures frequently present with multiple chronic diseases and complex antithrombotic regimens. Antiplatelet agents, vitamin K antagonists (VKAs), and direct oral anticoagulants (DOACs) are widely used to prevent arterial and venous thromboembolic events, while renal, hepatic, hematologic, and cardiovascular conditions may further complicate perioperative management (Bryant, 2022; de Oliveira et al., 2021; Soler-Espejo et al., 2023).

Postoperative bleeding is a recognized complication of invasive oral procedures, including tooth extraction, third-molar surgery, cyst surgery, osteotomy, and implant placement (Czembirek et al., 2014; Kiencało et al., 2021; Eichhorn et al., 2012; Hanken et al., 2016; Bajkin et al., 2009). Most bleeding episodes can be controlled with local measures, but clinically significant events may require emergency assessment or hospitalization (Czembirek et al., 2014; Eichhorn et al., 2012; Hanken et al., 2016; Bajkin et al., 2009; Clemm et al., 2016). Managing antithrombotic therapy therefore requires balancing bleeding-related concerns against the potentially serious consequences of treatment interruption.

During the study period, prescribing practice shifted from VKAs toward DOACs. These agents differ in indication, pharmacokinetics, monitoring requirements, and perioperative management (Soler-Espejo et al., 2023; Lin et al., 2021). Describing the medication distribution among patients hospitalized for bleeding may therefore provide useful clinical context, although such data cannot establish drug-specific bleeding rates without information on the number of exposed patients undergoing dental procedures.

Multimorbidity may create clinically distinct combinations of cardiovascular, metabolic, renal, hepatic, and vascular conditions. A person-centered method such as latent class analysis (LCA) can summarize recurring combinations of binary comorbidity indicators and quantify uncertainty in class membership (Whitson et al., 2016; Collins and Stephanie, 2013). This approach is more appropriate for identifying latent patient subgroups than applying principal component analysis to dichotomous diagnoses.

The present study aimed to characterize patients hospitalized for postoperative intraoral bleeding after dental or oral surgery at a single university center between 1 January 2011 and 31 December 2021. A secondary objective was to identify latent multimorbidity classes among patients with at least two documented comorbidities and to describe their demographic characteristics, clinical measurements, and observed antithrombotic medication distributions.

Because the cohort included only patients who had already experienced bleeding requiring hospitalization, the analysis was explicitly descriptive. It was not designed to identify predictors of bleeding, estimate procedure-specific or medication-specific bleeding risk, or support causal inference.

## 2 Materials and methods

### 2.1 Study population

This single-center retrospective study included inpatients treated in the Department of Oral and Maxillofacial Surgery at the University Hospital Schleswig-Holstein (UKSH), Campus Kiel, for intraoral bleeding complications following dental or oral surgery between 1 January 2011 and 31 December 2021. Patients treated exclusively as outpatients were not included. The study was approved by the local ethics committee (AZ D 474/21, Kiel).

### 2.2 Inclusion and exclusion criteria

Patients were included if they (1) presented with an anamnestically reported or clinically evident intraoral bleeding episode following a dental or oral surgical procedure and (2) required hospitalization because of the bleeding complication, irrespective of where the original procedure had been performed. The full descriptive cohort included patients aged 6–105 years. Patients managed exclusively in the outpatient setting were excluded.

### 2.3 Data collection and variable definitions

Study variables were collected by manual review of electronic medical records and paper-based clinical archives. For each admission, the indication for hospitalization, admission and discharge dates, clinical measurements, diagnoses, medication records, and documented management were extracted. Records were reviewed by two researchers, and discrepancies were resolved by consensus or consultation with a third reviewer.

Patient-level variables included age, sex, length of hospital stay, systolic and diastolic blood pressure at admission, hemoglobin, hematocrit, and international normalized ratio (INR). Medication categories were no antithrombotic medication, antiplatelet therapy, VKA, DOAC, antiplatelet therapy plus VKA, and antiplatelet therapy plus DOAC. INR was summarized for the overall cohort using observed admission values; the timing of measurement relative to any reversal treatment could not be determined consistently from the retrospective records, and INR was therefore interpreted descriptively only.

Antithrombotic medication use was ascertained through multiple chart sources, including active prescription records and medication order sheets at admission, pre-anesthetic assessment records, and attending physician progress notes. The type, dosage, and combination of antithrombotic agents, including VKA, DOAC, and antiplatelet agents, were systematically extracted from these records. Active prescriptions and medication order sheets served as the primary source, with progress notes used for supplementary verification. For latent class analysis, eleven binary comorbidity indicators were included: cardiac disease, hypertension, diabetes mellitus, renal disease, stroke/transient ischemic attack, deep venous thrombosis, heart valve replacement, peripheral arterial disease, gastrointestinal disease, rheumatic disease, and hepatic disease.

Comorbidities were selected from published literature on multimorbidity patterns and conditions relevant to bleeding or antithrombotic management and were coded as binary variables (0 = absent, 1 = present) (de Oliveira et al., 2021; Soler-Espejo et al., 2023; Whitson et al., 2016). They were identified from admission diagnoses, physician notes, and problem lists. Cardiac disease was based on a documented diagnosis or electrocardiographic report; hypertension on a documented diagnosis or antihypertensive treatment; diabetes mellitus on a documented diagnosis or antidiabetic treatment; renal disease on a documented diagnosis or elevated serum creatinine; stroke or transient ischemic attack, deep venous thrombosis, and heart valve replacement on documented neurological, vascular, or surgical history; and peripheral arterial disease, gastrointestinal disease, rheumatic disease, and hepatic disease on documented diagnoses in the medical record.

### 2.4 Statistical analysis

Latent class analysis was performed in 458 patients using 11 predefined binary comorbidity indicators. Models containing one to six classes were estimated with Mplus version 8.3 (Muthén & Muthén, Los Angeles, CA, USA) (Weller et al., 2020). Model selection considered the Akaike information criterion (AIC), Bayesian information criterion (BIC), sample-size-adjusted BIC (aBIC), entropy, bootstrap likelihood ratio test (BLRT), class size, and clinical interpretability, in accordance with established LCA guidance (Collins and Stephanie, 2013; Nylund et al., 2007). Lower information-criterion values indicate better relative fit, higher entropy indicates greater classification precision, and a significant BLRT supports a k-class model over a k-1-class model. Patients were assigned to the class with the highest posterior membership probability for descriptive comparisons.

After selection of the final model, demographic characteristics, clinical measurements, and observed antithrombotic medication categories were compared across latent classes. Continuous variables were compared using the Kruskal–Wallis test. Sex was compared using Pearson’s chi-square test. Because of small expected cell counts, differences in medication categories were evaluated using Fisher’s exact test with Monte Carlo simulation based on 10,000 replicates. All statistical tests were two-sided, and *P* values <0.05 were considered statistically significant.

The stability of the selected latent class solution was evaluated using 100 bootstrap resamples. Classification agreement between the original and bootstrap-derived solutions was assessed using the adjusted Rand index (ARI). Similarity of the latent class profiles was further evaluated using profile correlations and root mean square error (RMSE). Data processing and descriptive statistical analyses were performed using R version 4.2.2 (R Foundation for Statistical Computing, Vienna, Austria).

## 3 Results

### 3.1 Patient characteristics

The overall cohort comprised 700 patients hospitalized for postoperative intraoral bleeding following dental or oral surgery. The median age was 74 years (IQR 65–80), and 460 patients (65.7%) were male. Median hospital stay was 2 days (IQR 1–3). Cardiac disease was documented in 524 patients (74.9%), hypertension in 336 (48.0%), diabetes mellitus in 123 (17.6%), heart valve replacement in 97 (13.9%), and renal disease in 96 (13.7%). A total of 581 patients (83.0%) were receiving at least one antithrombotic medication at admission. Medication categories were VKA monotherapy in 317 patients (45.3%), DOAC monotherapy in 124 (17.7%), antiplatelet monotherapy in 95 (13.6%), antiplatelet therapy plus DOAC in 23 (3.3%), antiplatelet therapy plus VKA in 22 (3.1%), and no antithrombotic medication in 119 (17.0%) (Table 1).

**TABLE 1:** Demographic and clinical characteristics of the overall study cohort.

Characteristic	Overall (N = 700)
Age, years, median (IQR)	74 (65–80) [n = 700]
Length of hospital stay, days, median (IQR)	2.00 (1.00–3.00) [n = 700]
Systolic blood pressure, mmHg, median (IQR)	130 (120–140) [n = 527]
Diastolic blood pressure, mmHg, median (IQR)	80 (70–80) [n = 527]
Hemoglobin, g/dL, median (IQR)	12.9 (11.5–14.1) [n = 642]
Hematocrit, %, median (IQR)	37.5 (33.7–41.0) [n = 642]
International normalized ratio, median (IQR)	1.28 (1.11–1.76) [n = 629]
Sex, n (%)	​
Male	460 (65.7%)
Female	240 (34.3%)
Cardiac disease, n (%)	524 (74.9%)
Renal disease, n (%)	96 (13.7%)
Hepatic disease, n (%)	18 (2.6%)
Rheumatic disease, n (%)	39 (5.6%)
Gastrointestinal disease, n (%)	77 (11.0%)
Stroke, n (%)	75 (10.7%)
Hypertension, n (%)	336 (48.0%)
Diabetes mellitus, n (%)	123 (17.6%)
Peripheral arterial disease, n (%)	13 (1.9%)
Deep venous thrombosis, n (%)	31 (4.4%)
Heart valve replacement, n (%)	97 (13.9%)
Antithrombotic medication, n (%)	​
No medication	119 (17.0%)
Antiplatelet therapy	95 (13.6%)
DOAC	124 (17.7%)
VKA	317 (45.3%)
Antiplatelet therapy + DOAC	23 (3.3%)
Antiplatelet therapy + VKA	22 (3.1%)

Data are presented as median (interquartile range) [available n] for continuous variables and n (%) for categorical variables. DOAC, direct oral anticoagulant; INR, international normalized ratio; VKA, vitamin K antagonist.

### 3.2 Changes in antithrombotic medication categories over time

The annual distribution of antithrombotic medication categories among the 700 hospitalized bleeding cases is presented in Table 2. VKA monotherapy accounted for the largest number of cases in the earlier years, whereas the number of cases involving DOACs increased after their introduction. DOAC monotherapy cases increased from none in 2011 to 25 in 2020, while VKA monotherapy cases declined from 36 in 2011 to seven in 2021. Combined antiplatelet and VKA therapy was documented from 2011 to 2016 but not thereafter. These are counts among hospitalized cases and should not be interpreted as medication-specific bleeding rates.

**TABLE 2:** Annual distribution of antithrombotic medication categories among hospitalized bleeding cases.

Year of inpatient treatment	Antiplatelet therapy	Antiplatelet therapy + DOAC	No medication	DOAC	VKA	Antiplatelet therapy + VKA
2011	8	0	16	0	36	3
2012	8	0	17	1	28	2
2013	11	0	15	4	49	5
2014	3	2	11	6	48	3
2015	8	2	11	11	40	4
2016	10	5	15	13	29	5
2017	8	0	10	15	24	0
2018	9	6	5	21	29	0
2019	10	1	11	19	13	0
2020	11	6	5	25	14	0
2021	9	1	3	9	7	0

### 3.3 Latent class model selection and class profiles

Of the 700 patients in the overall cohort, 458 had at least two documented comorbidities and were included in the latent class analysis. Models containing one to six latent classes were evaluated using 11 binary comorbidity indicators. The fit indices for the candidate models are presented in Table 3.

**TABLE 3:** Fit indices for one-to six-class latent class models based on 11 binary comorbidity indicators.

Number of classes	Log-likelihood	AIC	BIC	aBIC	Entropy	BLRT	Class sizes (%)
1	−1919.715	3861.430	3906.825	3871.914	​	​	100%
2	−1880.277	3806.554	3901.472	3828.477	1.000	<0.001	78.82%/21.18%
3	−1856.720	3783.441	3927.881	3816.802	0.754	<0.001	39.08%/21.18%/39.74%
4	−1836.334	3766.669	3960.632	3811.468	0.798	<0.001	35.59%/35.37%/20.52%/8.52%
5	−1820.781	3759.562	4003.048	3815.800	0.871	<0.001	5.68%/21.18%/12.01%/32.97%/28.17%
6	−1809.665	3761.330	4054.338	3829.005	0.823	0.667	5.68%/19.65%/21.18%/12.01%/8.73%/32.75%

AIC, akaike information criterion; aBIC, sample-size-adjusted Bayesian information criterion; BIC, bayesian information criterion; BLRT, bootstrap likelihood ratio test. Lower AIC, BIC, and aBIC, values indicate better relative fit, whereas higher entropy indicates greater classification precision. Model selection considered statistical fit, entropy, BLRT, results, class size, and clinical interpretability.

The five-class model had the lowest AIC (3759.562), entropy of 0.871, and a significant BLRT compared with the four-class model (*P* < 0.001). The BIC favored the two-class model and the aBIC favored the four-class model, demonstrating that model-selection indices did not uniformly identify the same solution. The six-class model had higher AIC, BIC, and aBIC values, lower entropy, and a nonsignificant BLRT (*P* = 0.667). The five-class model was retained after considering the combined statistical evidence, classification precision, class size, and the objective of distinguishing clinically interpretable multimorbidity profiles. This selection should nevertheless be interpreted in light of the disagreement among information criteria and the moderate bootstrap stability reported below.

Class-specific conditional probabilities for the 11 comorbidity indicators are shown in Figure 1. Class 1, the venous thrombotic-cardiac class, was characterized by deep venous thrombosis together with cardiac disease and hypertension. Class 2, the valvular-cardiac class, was distinguished by heart valve replacement and cardiac disease. Class 3, the cerebrovascular-cardiac class, was characterized by stroke together with cardiac disease and hypertension. Class 4, the hypertensive-cardiac class, showed prominent hypertension and cardiac disease. Class 5, the multisystem cardiometabolic class, had a broader profile involving cardiac disease, hypertension, diabetes mellitus, renal disease, and gastrointestinal disease. The class labels are descriptive summaries of the highest conditional probabilities and are not intended to represent validated clinical phenotypes.

**FIGURE 1:**
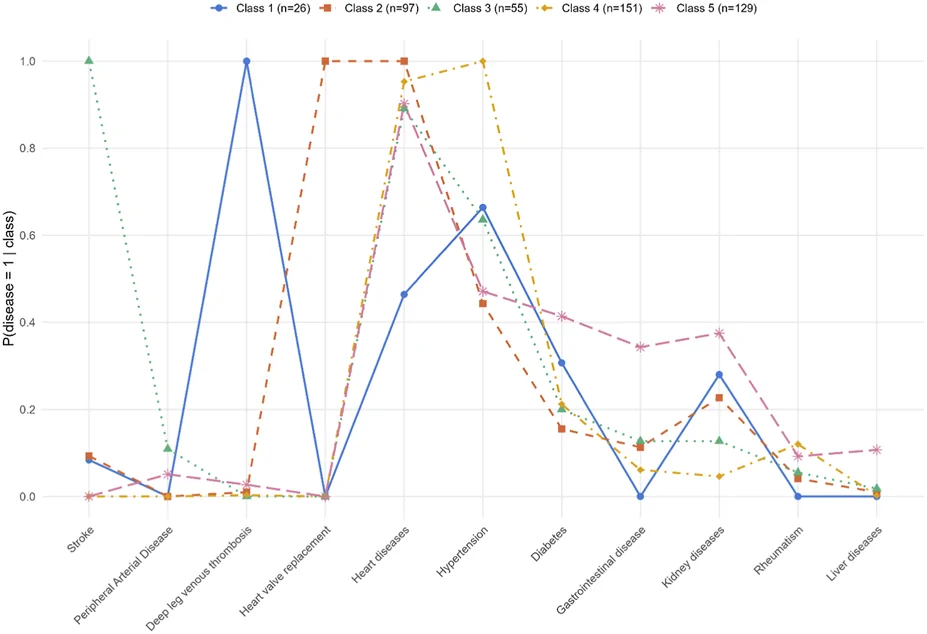
Conditional item-response probability profiles for the selected five-class latent class model. Notes: The y-axis represents the estimated probability of each comorbidity being present conditional on latent class membership.

### 3.4 Medication characteristics across the latent classes

The observed distribution of antithrombotic medication categories differed across the five assigned latent classes (Fisher exact test with Monte Carlo simulation, *P* < 0.001; Table 4). VKA monotherapy was most frequent in Classes 1 and 2, accounting for 73.1% and 77.3% of patients, respectively. In Classes 3–5, VKA monotherapy ranged from 43.6% to 48.3%. DOAC monotherapy was most frequent in Class 4 (25.8%), followed by Class 3 (23.6%) and Class 5 (21.7%). Antiplatelet monotherapy was more frequent in Classes 3–5 than in Classes 1 and 2. These distributions describe medication exposure among hospitalized bleeding cases.

**TABLE 4:** Observed antithrombotic medication distributions across the five assigned latent classes.

Observed antithrombotic medication	Class 1 (n = 26)	Class 2 (n = 97)	Class 3 (n = 55)	Class 4 (n = 151)	Class 5 (n = 129)	*P* value
Observed antithrombotic medication, n (%)						
No medication, n (%)	1 (3.8%)	0 (0.0%)	0 (0.0%)	9 (6.0%)	8 (6.2%)	<0.001
Antiplatelet therapy, n (%)	1 (3.8%)	5 (5.2%)	9 (16.4%)	23 (15.2%)	22 (17.1%)	​
DOAC, n (%)	2 (7.7%)	12 (12.4%)	13 (23.6%)	39 (25.8%)	28 (21.7%)	​
VKA, n (%)	19 (73.1%)	75 (77.3%)	24 (43.6%)	73 (48.3%)	61 (47.3%)	​
Antiplatelet therapy + DOAC, n (%)	0 (0.0%)	4 (4.1%)	5 (9.1%)	5 (3.3%)	5 (3.9%)	​
Antiplatelet therapy + VKA, n (%)	3 (11.5%)	1 (1.0%)	4 (7.3%)	2 (1.3%)	5 (3.9%)	​

### 3.5 Sensitivity and stability analyses

Age, sex, length of hospital stay, comorbidity indicators, and antithrombotic medication categories were complete. Systolic and diastolic blood pressure were each missing for 173 patients (24.7%), hemoglobin and hematocrit for 58 (8.3%), and INR for 71 (10.1%); no values were imputed (Supplementary Table S1). All 100 bootstrap resamples were successfully estimated. The mean adjusted Rand index was 0.607 (standard deviation 0.158; median 0.615), the mean class-profile correlation was 0.871, and the mean profile RMSE was 0.130. Class-specific profile correlations were 0.741, 0.963, 0.786, 0.991, and 0.875 for Classes 1–5, respectively. These findings indicate moderate overall stability, with greater profile stability for Classes 2 and 4 and lower stability for Classes 1 and 3 (Supplementary Table S2).

## 4 Discussion

This retrospective study characterized 700 patients hospitalized for postoperative intraoral bleeding following dental or oral surgery and identified five latent multimorbidity classes among 458 patients with at least two documented comorbidities. The cohort was predominantly older and medically complex, 83.0% were receiving at least one antithrombotic medication, and the observed medication distribution changed over the 2011–2021 study period. The LCA further described heterogeneity in combinations of cardiovascular, vascular, metabolic, renal, and gastrointestinal conditions.

The findings must be interpreted as descriptive characteristics of patients who had already experienced bleeding requiring hospitalization. Because the study did not include a comparator group of patients undergoing similar procedures without bleeding, it cannot determine whether a medication, comorbidity, or latent class increases the risk of postoperative bleeding or hospitalization. Likewise, annual counts of VKA- and DOAC-associated admissions cannot be converted into drug-specific rates because the numbers of exposed patients and dental procedures in the source population were unavailable.

The temporal distribution is nevertheless consistent with the broader transition from VKAs toward DOACs for non-valvular atrial fibrillation and venous thromboembolism (Barnes et al., 2023; Bejjani et al., 2024; Warkentin et al., 2022). VKA monotherapy accounted for more hospitalized cases in the earlier years, while DOAC-associated cases became more frequent later.

The five latent classes summarized recurring combinations of binary comorbidities. Classes 1 and 2 were strongly characterized by venous thrombotic or valvular cardiac conditions and had the highest proportions of VKA use, whereas Class 4 had the highest proportion of DOAC use. These medication distributions are clinically plausible because underlying indications influence antithrombotic selection.

Model selection required balancing several criteria. Although the five-class model had the lowest AIC, high entropy, and a significant BLRT, the BIC favored two classes and the aBIC favored four classes. Bootstrap validation showed moderate rather than high overall agreement, and profile stability was lower for Classes 1 and 3. The five-class solution should therefore be considered an exploratory description that requires external replication.

The admission INR median of 1.28 was calculated from 629 observed values across the entire cohort, including patients not receiving VKAs. It is therefore not a VKA-specific therapeutic-level estimate. In addition, the timing of INR measurement relative to any reversal treatment could not be determined consistently. The INR result is reported only as a descriptive admission measurement and should not be used to infer anticoagulation intensity or treatment quality.

For clinical practice, the data reinforce the complexity of hospitalized bleeding cases and the need to review comorbidities, antithrombotic indications, procedure characteristics, and local hemostatic options together. Existing evidence generally supports continuation of antithrombotic therapy for many minor oral procedures with appropriate local hemostasis, while individualized and interdisciplinary planning remains important for complex procedures or patients with high thromboembolic risk (Kämmerer et al., 2015; Dou et al., 2025; Ockerman et al., 2021; Curtis et al., 2025; Girotra et al., 2014; Yong et al., 2022; Dawoud et al., 2021; Madrid and Sanz, 2009). The present study did not test any specific perioperative strategy.

This study has several limitations. The cohort contained only hospitalized bleeding cases, without a non-bleeding comparator or denominator of procedures and medication exposure. The LCA was restricted to 458 patients with at least two documented comorbidities, model-selection indices were discordant, and bootstrap stability was moderate. Class assignment also simplifies posterior membership uncertainty.

The study also has strengths, including a relatively large hospitalized cohort spanning 11 years, systematic chart review, use of a probabilistic method appropriate for binary comorbidity indicators, transparent comparison of one-to six-class models, explicit reporting of missingness without imputation, and bootstrap assessment of class stability.

Overall, the results provide a descriptive account of multimorbidity and antithrombotic medication distributions among patients hospitalized for postoperative dental bleeding. Future studies should include both bleeding and non-bleeding patients, reliable procedural denominators, standardized timing of laboratory measurements, and external validation of any proposed multimorbidity classes before evaluating their prognostic or clinical utility.

## 5 Conclusion

Among 700 patients hospitalized for postoperative intraoral bleeding, most were older, medically complex, and receiving antithrombotic medication. In the subgroup with at least two documented comorbidities, LCA identified five exploratory multimorbidity classes with different observed antithrombotic medication distributions.

These findings characterize heterogeneity among hospitalized bleeding cases but do not establish independent risk factors, medication-specific bleeding rates, causal relationships, or individualized risk predictions. The selected five-class solution showed moderate bootstrap stability and requires replication in independent cohorts.

Future comparative studies should include patients with and without postoperative bleeding and incorporate procedure-level denominators, medication exposure, perioperative management, and standardized outcome definitions.

## Data Availability

The raw data supporting the conclusions of this article will be made available by the authors, without undue reservation.
